# A model for estimating the brainstem volume in normal healthy individuals and its application to diffuse axonal injury patients

**DOI:** 10.1038/s41598-022-27202-x

**Published:** 2023-01-02

**Authors:** Gaku Fujimoto, Shiho Ubukata, Genichi Sugihara, Naoya Oishi, Toshihiko Aso, Toshiya Murai, Keita Ueda

**Affiliations:** 1 Department of Psychiatry, Graduate School of Medicine, Kyoto University, 54 Shogoin-Kawahara-Cho, Sakyo-Ku, Kyoto, 606-8507 Japan; 2 Medical Innovation Center, Graduate School of Medicine, Kyoto University, 53 Shogoin-Kawahara-Cho, Sakyo-Ku, Kyoto, 606-8507 Japan; 3 Department of Psychiatry and Behavioral Sciences, Graduate School of Medical and Dental Sciences, Tokyo Medical and Dental University, 1-5-45 Yushima, Bunkyo-Ku, Tokyo, 113-8510 Japan; 4 Laboratory for Brain Connectomics Imaging, RIKEN Center for Biosystems Dynamics Research, 6-7-3 Minatojima-Minamimachi, Chuo-Ku, Kobe, Hyogo 650-0047 Japan; 5 Department of Medical Welfare, Faculty of Health Sciences, Kyoto Koka Women’s University, 38 Nishikyogoku Kadono-Cho, Ukyo-Ku, Kyoto, 615-0882 Japan

**Keywords:** Brain injuries, Magnetic resonance imaging, Neurodegeneration

## Abstract

Diffuse axonal injury (DAI) is a subtype of traumatic brain injury that causes acute-phase consciousness disorders and widespread chronic-phase brain atrophy. Considering the importance of brainstem damage in DAI, a valid method for evaluating brainstem volume is required. We obtained volume measurements from 182 healthy adults by analyzing T1-weighted magnetic resonance images, and created an age-/sex-/intracranial volume-based quantitative model to estimate the normal healthy volume of the brainstem and cerebrum. We then applied this model to the volume measurements of 22 DAI patients, most of whom were in the long-term chronic phase and had no gross focal injury, to estimate the percentage difference in volume from the expected normal healthy volume in different brain regions, and investigated its association with the duration of posttraumatic amnesia (which is an early marker of injury severity). The average loss of the whole brainstem was 13.9%. Moreover, the percentage loss of the whole brainstem, and particularly of the pons and midbrain, was significantly negatively correlated with the duration of posttraumatic amnesia. Our findings suggest that injury severity, as denoted by the duration of posttraumatic amnesia, is among the factors affecting the chronic-phase brainstem volume in patients with DAI.

## Introduction

Traumatic brain injury (TBI) is a major cause of disability, especially among young people, and is a critical health and socioeconomic problem worldwide^1^. Some patients with TBI have gross focal injury (FI), which is characterized by a large focal lesion caused by an external force, whereas others have no visible FI. The latter TBI cases likely exhibit diffuse pathology, and diffuse axonal injury (DAI) is a major clinical entity of this type^2^. DAI is considered a primary cause of consciousness disorder in acute-phase TBI^3^.

DAI leads to post-acute-phase widespread brain atrophy^4–7^, which results from Wallerian degeneration and delayed neuronal cell death, following the process of primary and secondary axotomy because of axonal injury^7^. Histopathological observations have revealed that axonal injury in DAI is not evenly diffuse throughout the brain; rather, it is concentrated in specific brain regions, one of which is the brainstem^2,8–11^. Given that axonal injury in the brainstem is a primary factor in the development of immediate consciousness disorder in DAI or TBI^11–13^, the post-acute-phase volume of the brainstem in DAI is of clinical interest because it likely reflects the severity of acute-phase consciousness disorder. However, this issue has not been extensively investigated. Post-TBI brainstem volume reduction has been reported previously in studies by Kim et al.^14^ and Cole et al.^15^; however, they used tensor-based morphometry or whole-brain voxel-based morphometry analyses in patients with mild or moderate-severe TBI. Therefore, there is currently limited research on patients across all levels of TBI severity that focuses on the brainstem or its subfields as regions of interest (ROIs). Warner et al.^16^ reported volume reductions in the brainstem of patients with traumatic axonal injury, although the exclusion criteria for FI were relatively liberal (i.e., any focal, mixed, or high-density lesion > 10 ml). Therefore, brainstem volume changes that are attributed solely to axonal injury have not been fully examined. Brezova et al.^17^ and Sidaros et al.^5^ also reported brainstem volume reduction post-injury; however, the time since injury in the investigated subjects was < 600 days, when atrophy caused by TBI may still have been in progress^18^. Taken together, there is insufficient information on the volume of the brainstem (either the whole brainstem or its subfields) long after injury or its association with initial injury severity. As such, there are limited normative data available on the volume of the whole brainstem or any of its subfields. The possibility that the conventional intracranial volume (ICV) divisor correction or proportions method^19,20^ is not strictly suitable for brainstem volumetric measures cannot be ignored. Thus, a new method for evaluating the brainstem volume of any individual in relation to the healthy volume would be desirable for analysis purposes.

In the present study, we investigated the brainstem volumes of healthy individuals using the following approach. In Experiment 1, we measured the brainstem volumes of a large number of healthy adults and established a model to estimate the “expected normal healthy volume” (ENHV) of the brainstem in any individual based on the ICV, age, and sex. In Experiment 2, the first attempt to apply the model to a non-healthy sample was made; we estimated the percentage difference between the actual brainstem volume and ENHV, and investigated the association of the ENHV-adjusted brainstem volume with acute-phase injury severity in DAI subjects (who were mainly in the long-term chronic phase and had no gross FI). The acute-phase severity of TBI is generally taken to indicate the extent of consciousness disorder, which can be measured by clinical scales such as the Glasgow Coma Scale (GCS) and Japan Coma Scale (JCS), or as the duration of posttraumatic amnesia (PTA). PTA is a state of confusion that occurs immediately after TBI, in which the patient is disoriented and unable to recall new memories^21–23^. In this study, the duration of PTA, which was obtained for every DAI subject, was employed in the analysis as an indicator of injury severity. We hypothesized that a chronic volume reduction would be seen in the brainstem, and that this reduction would be associated with the severity of the acute injury. It should be noted that, considering the global atrophy of the brain that occurs in DAI, we used the whole cerebrum as a control ROI in all analyses of brainstem volume.

## Results

### Experiment 1

#### Participants

In total, 185 and 47 healthy subjects participated in Experiment 1, and three of the 185 healthy subjects were excluded from the analysis because of image processing errors. Finally, the data of 182 healthy subjects (Group A) and 47 healthy subjects (Group B) were included in the analysis. There was no significant difference between Group A and B in age or sex. Table 1 shows the demographic characteristics of the participants.

**Table 1:** Demographic characteristics of the participants in Experiment 1.

Characteristics	Group A (n = 182)	Group B (n = 47)	*P-*value
Age^a^ (years)	33 (18–64)	34 (20–68)	0.560^b^
Sex (male/female)	99/83	24/23	0.683^c^

^a^Median (range).

^b^Groups were compared with the Mann–Whitney U test.

^c^Groups were compared with the chi-square test.

#### Creation of the model for estimating ENHV

**Figure 1:**
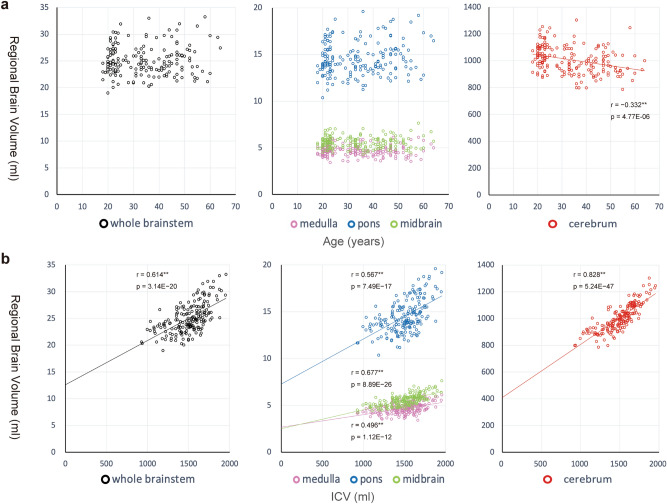
Relationship between each ROI volume and age (a) or ICV (b) in 182 healthy subjects. **(a**) There were no significant correlations between regional volume and age for the whole brainstem (Pearson’s r = 0.093, *P *= 0.213), medulla (r = 0.029, *P *= 0.698), pons (r = 0.145, *P *= 0.050), or midbrain (r = 0.00046, *P *= 0.995), whereas a significant negative correlation was found for the cerebrum (r =  − 0.332, P = 4.77E-06). **(b**) There were significant positive correlations between regional volume and ICV (r = 0.496, 0.567, 0.677, 0.614, and 0.828 for the medulla, pons, midbrain, whole brainstem, and cerebrum, respectively; P < 1.0E-11). However, none of the ROI volumes showed direct proportionality to ICV, reflected by the positive y-intercepts of the regression lines on the scatterplot with ICV on the x-axis and each regional volume on the y-axis. *Significant at *P *< 0.05. *ROI* Region of interest, *ICV* Intracranial volume.

Figure 1 shows the relationship between each regional brain volume and age or ICV in the healthy subjects of Group A. There was no significant correlation between age and regional volume of the whole brainstem (r = 0.093, *P *= 0.213), medulla (r = 0.029, *P *= 0.698), pons (r = 0.145, *P *= 0.050), or midbrain (r = 0.00046, *P *= 0.995). However, there was a significant negative correlation between age and the volume of the cerebrum (r =  − 0.332, *P *= 4.77E-06; Fig. 1a). The volume of each ROI showed a significant positive correlation with ICV (r = 0.496, 0.567, 0.677, 0.614, and 0.828 for the medulla, pons, midbrain, whole brainstem, and cerebrum, respectively; *P *< 1.0E − 11; Fig. 1b). Two-way ANOVA revealed significant interactions between sex and ICV for the volume of the whole brainstem (F[1, 178] = 8.46, P = 0.004), midbrain (F[1, 178] = 6.49, P = 0.012), pons (F[1, 178] = 8.60, P = 0.004), and cerebrum (F[1, 178] = 7.11, P = 0.008) but not the medulla (F[1, 178] = 3.44, *P *= 0.065). Single/multiple regression analyses were performed for each sex group for all ROIs except the medulla, which was analyzed with the two sex groups combined. The explanatory variables were ICV and age for the cerebrum and only ICV for the other four ROIs (Table 2). The results of the regression analyses are shown in Supplementary Table 1, and according to the obtained regression equations, the model equations were fixed as Eqs. (1), (2), and (3) in Table 2, with the corresponding coefficients and the dummy variable representing sex shown in Table 3.

**Table 2:** Sample and explanatory variables of regression analysis and the model equation form.

ROI	Sample for regression analysis	Explanatory variables	Forms of the model equations	
Medulla	Whole sample (two sexes together) of Group A	ICV	${\text{ENHV }} = a \times ICV + b$	(1)
Whole brainstem	Each sex subgroup of Group A	ICV	${\text{ENHV }} = ({1} - sex) \, \times \, (a_{f} \times \, ICV + b_{f} ) \, + sex \, \times \, (a_{m} \times \, ICV + b_{m} )$	(2)
Pons				
Midbrain				
Cerebrum	Each sex subgroup of Group A	ICV and age	${\text{ENHV }} = \, ({1} - sex) \, \times \, (a_{f} \times \, ICV + b_{f} \times \, age + c_{f} ) + sex \times \, (a_{m} \times \, ICV + b_{m} \times \, age + c_{m} )$	(3)

Each ROI and its corresponding model equation for estimating healthy volume are shown. The equations had three forms for the five ROIs. _“_$a_{f} \times \, ICV + b_{f}$_”_ or _“_$a_{f} \times \, ICV + b_{f} \times \, age + c_{f}$_”_ is the regression equation obtained in the regression analysis for the female subgroup, and “$a_{m} \times \, ICV + b_{m}$” or “$a_{m} \times \, ICV + b_{m} \times \, age + c_{m}$” is that for the male subgroup.

*sex* = 1 for males/0 for females in Eqs. (2) and (3).

*ROI* Region of interest, *ICV* Intracranial volume, *ENHV* Expected normal healthy volume.

**Table 3:** Coefficients of the model equations.

**Coefficients in Equation (1)**						
	*a*	*b*				
Medulla	1.35E−03	2.65E + 03				
**Coefficients in Equation (2)**						
	*a*_*f*_	*b*_*f*_	*a*_*m*_	*b*_*m*_		
Whole brainstem	3.92E−03	1.80E + 04	9.50E−03	1.10E + 04		
Pons	2.01E−03	1.08E + 04	5.74E−03	6.06E + 03		
Midbrain	1.19E−03	3.55E + 03	2.20E−03	2.30E + 03		
**Coefficients in Equation (3)**						
	*a*_*f*_	*b*_*f*_	*c*_*f*_	*a*_*m*_	*b*_*m*_	*c*_*m*_
Cerebrum	2.90E−01	− 1.54E + 03	6.03E + 05	4.41E−01	− 2.01E + 03	4.11E + 05

This table should be used with Eqs. (1), (2), and (3) in Table 2.

#### Model validation

The intraclass correlation coefficients (ICCs) obtained from the reliability test of the ROIs are shown in Table 4. For all ROIs, the ICC was > 0.4. Thus, our model had sufficient validity for application of all five ROIs to Japanese and East Asian adults.

**Table 4:** Reliability of the model tested on 47 healthy adults from Group B.

ROI	Intraclass correlation coefficient (95% CI)	*P-*value
Whole brainstem	0.715 (0.540–0.830)	5.8E−10
Medulla	0.548 (0.312–0.720)	2.8E−05
Pons	0.657 (0.458–0.793)	2.0E−07
Midbrain	0.723 (0.552–0.836)	3.3E−09
Cerebrum	0.922 (0.864–0.956)	< 1.0E−10

Intraclass correlation coefficients, calculated using a two-way mixed effects model for single measures (absolute agreement), were used to assess the reliability of the model, i.e., the correlations between measured and corresponding expected normal healthy volumes.

*ROI* Region of interest, *CI* Confidence interval.

### Experiment 2

#### Participants

Twenty-two DAI patients and 60 healthy controls (HCs) participated in Experiment 2. There was no significant difference between the DAI patients and HCs in age or sex. The demographic and clinical characteristics of the participants are shown in Table 5. Clinical details of the DAI patients are shown in Supplementary Table 2.

**Table 5:** Demographic and clinical characteristics of the participants in Experiment 2.

Characteristics	DAI patients (n = 22)	Healthy controls (n = 60)	*P-*value
Age^a^ (years)	36 (21–61)	33.5 (22–64)	0.593^b^
Sex (male/female)	17/5	47/13	1.000^c^
Time since injury^a^ (months)	53 (5–355)		
Cause of injury (n)	Traffic accident, n = 21; fall, n = 1		
Anatomical grading for DAI^d^ (persons)	Stage I, n = 8; stage II, n = 4; stage III, n = 3; stage IV, n = 2; stage 0, n = 4; NA, n = 1		
GCS score^a^	10 (3–15) (n = 11)		
JCS score^a^	200 (2–300) (n = 7)		
PTA^a, e^ (weeks)	7.5 (1–26)		
Injury severity by GCS or JCS (n)	severe 11, moderate 2, mild 5, undetermined 4		

^a^Median (range).

^b^Groups were compared with the Mann–Whitney U test.

^c^Groups were compared with the chi-square test.

^d^Anatomical grading for DAI: stage I, hemispheric lesions; stage II, hemispheric and additional corpus callosum lesions; stage III, brainstem lesions; stage IV, lesions in the substantia nigra or mesencephalic tegmentum; stage 0, absence of hemorrhagic spots in chronic-phase images but evidence of microbleeds on acute-phase X-ray computed tomography images.

^e^ “PTA (weeks) = X” indicates “7X–6 ≤ PTA (days) ≤ 7X”. For example, “PTA (weeks) = 2” indicates “PTA (days) = 8–14”.

*DAI* Diffuse axonal injury, *GCS* Glasgow coma scale, *JCS* Japan coma scale, *PTA* Duration of posttraumatic amnesia, *NA* Not available.

#### Group comparisons: 22 DAI patients vs. 60 HCs

The ICVs and volumes of the five ROIs for each group are shown in Table 6. Mann–Whitney U tests revealed significant differences in volume between the two groups for all regions except for ICV, and all survived Bonferroni correction for multiple comparisons.

**Table 6:** ICV and volumes of the five ROIs in DAI patients and healthy controls.

Type of volume	Region	DAI (*n* = 22)	Healthy (*n* = 60)	*P-*value^a^
Measured volume^b^	ICV	(15.64 ± 1.77) E + 05	(15.44 ± 1.92) E + 05	0.90
	Whole brainstem	(22.10 ± 2.81) E + 03	(25.75 ± 2.51) E + 03	4.34E−06*
	Medulla	(43.57 ± 4.87) E + 02	(47.86 ± 5.78) E + 02	3.27E−03*
	Pons	(12.48 ± 1.67) E + 03	(14.93 ± 1.58) E + 03	7.41E−07*
	Midbrain	(50.15 ± 7.88) E + 02	(57.39 ± 5.30) E + 02	2.25E−05*
	Cerebrum	(94.77 ± 6.98) E + 04	(101.87 ± 9.32) E + 04	1.31E−03*
ENHV-adjusted volume (Percent of ENHV)	Whole brainstem	86.1 ± 11.4	100.9 ± 8.1	3.65E−07*
	Medulla	91.1 ± 10.2	100.8 ± 10.8	5.75E−04*
	Pons	83.7 ± 11.6	100.8 ± 9.2	2.48E−07*
	Midbrain	88.1 ± 14.8	101.4 ± 7.3	1.28E−05*
	Cerebrum	92.6 ± 7.2	99.9 ± 4.3	1.40E−06*

^a^Groups were compared with the Mann–Whitney U test.

^b^The unit of volume is mm^3^.

*Significant at *P *< 0.05 with Bonferroni correction.

*ICV* Intracranial volume, *ROI* Region of interest, *DAI* Diffuse axonal injury, *ENHV* Expected normal healthy volume.

#### ENHV-adjusted volume of each ROI in DAI patients

The descriptive statistics of the ENHV-adjusted volume for each ROI for both groups are shown in Table 6. In the DAI patients, the ENHV-adjusted volume was significantly smaller in the whole brainstem (86.1% ± 11.4%) than that in the cerebrum (92.6% ± 7.2%, related-samples Wilcoxon signed-rank test, P = 7.79E−04). The Friedman test showed significant differences in the ENHV-adjusted volume among the three subfields of the brainstem (P = 6.15E−03), and the related-samples Wilcoxon signed-rank test revealed significant differences between the pons and midbrain (P = 1.93E−03) and between the pons and medulla (P = 1.38E−03), even after Bonferroni correction for multiple comparisons. However, there was no significant difference between the midbrain and medulla (P = 1.40E−01). In DAI patients, the pons had a significantly greater ratio of volume loss than that in the midbrain or medulla.

We confirmed that the mean ENHV-adjusted volume in HCs was reasonably close to 100 (98.5–101.5) for all ROIs.

#### Duration of PTA and volume of each ROI in DAI patients

**Figure 2:**
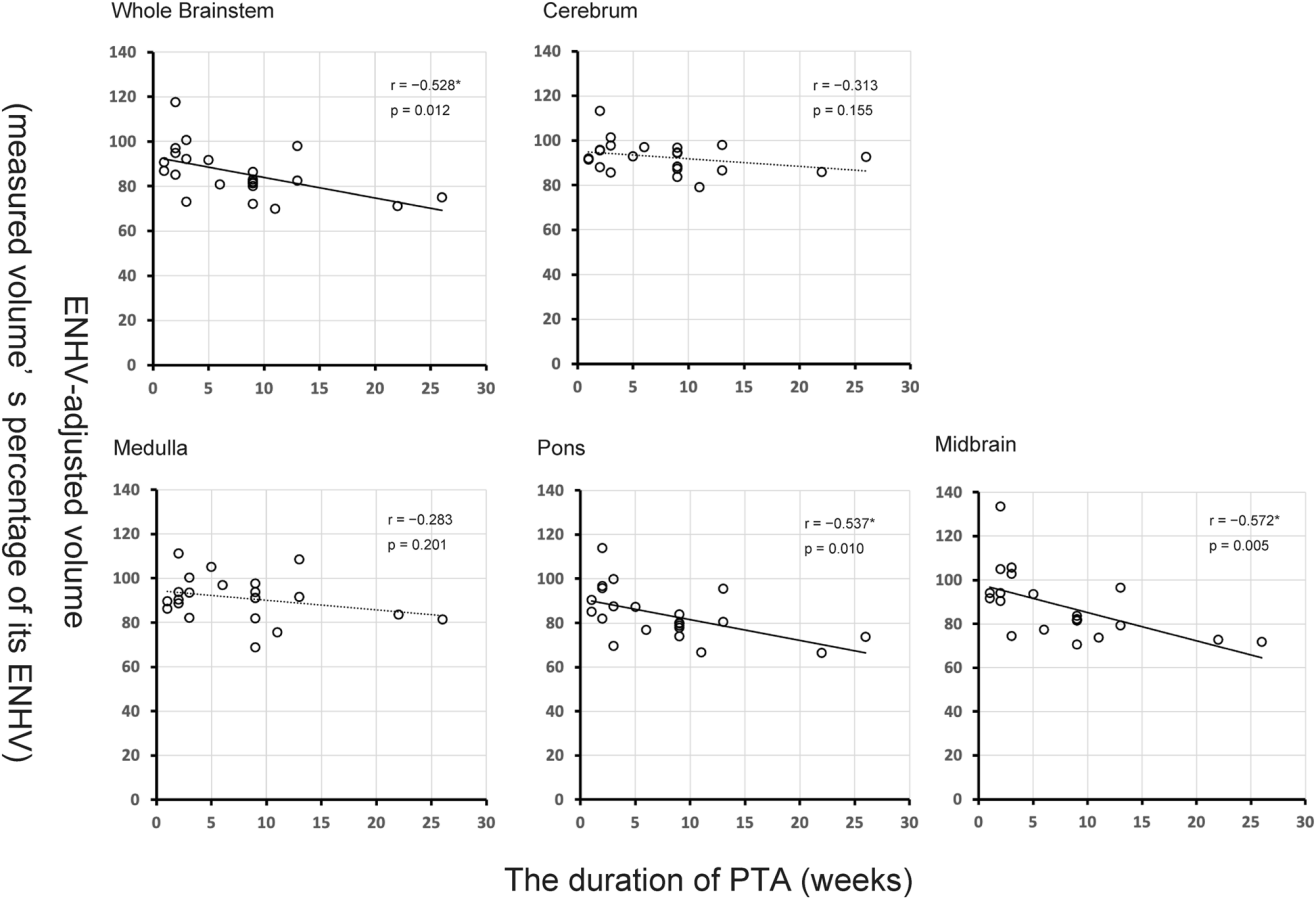
The duration of PTA and ENHV-adjusted volume in 22 DAI patients. The negative correlation between the duration of PTA and ENHV-adjusted volume was significant at *P *< 0.05 in the whole brainstem (r =  − 0.528, *P *= 0.012), pons (r =  − 0.537, *P *= 0.010), and midbrain (r =  − 0.572, *P *= 0.005) but not in the cerebrum (*r* =  − 0.313, *P *= 0.155) or medulla (r =  − 0.283, *P *= 0.201). *Significant at *P *< 0.05. *PTA* Posttraumatic amnesia, *ENHV* Expected normal healthy volume, *DAI* Diffuse axonal injury.

Scatterplots of the duration of PTA and ENHV-adjusted volumes of the 22 DAI patients for each ROI are shown in Fig. 2 with respective Pearson’s r- and *P-*values. Negative correlations between the duration of PTA and ENHV-adjusted volume were significant at *P *< 0.05 (uncorrected) in the whole brainstem (r =  − 0.528, *P *= 0.012), pons (r =  − 0.537, *P *= 0.010), and midbrain (r =  − 0.572, *P *= 0.005) but not in the cerebrum or medulla. The partial correlation analysis, controlling for time since injury, also yielded a similar result, which confirmed the significant negative correlations in the whole brainstem (r =  − 0.449, *P *= 0.041), pons (r =  − 0.459, *P *= 0.036), and midbrain (r =  − 0.502, *P *= 0.020; Supplementary Table 3). In contrast, the partial correlation analyses where the ENHV-adjusted volume was replaced with the conventional ICV-corrected volume revealed a significant association in only the midbrain (r =  − 0.446, *P *= 0.042).

## Discussion

This study investigated chronic-phase DAI subjects and demonstrated (i) marked volume reduction in the brainstem and (ii) a negative correlation between the duration of PTA and brainstem volume. An advantage of this study is the use of volume adjustment. Because brain volume varies among normal healthy individuals depending on ICV, age, and sex, we adjusted each regional brain volume for these variables using a large magnetic resonance imaging (MRI) dataset of normal healthy subjects.

The ICV measures in this study were comparable with those reported in previous studies^24,25^. Our volume measures of the brainstem (and its subfields) of healthy participants agree well with those presented by Sander et al.^26^ Moreover, we did not observe brainstem volume reductions with increasing age, which was also consistent with previous studies^26–28^. Our method—representing a volume of a particular ROI of an individual subject as a value of the raw volume divided by the estimated volume of non-brain-injured healthy individuals with the same ICV, age, and sex—has an advantage over the proportions method (i.e., volume divided by ICV). Although ICV correction using the proportions method is widely used, there is no guarantee that the proportionality between the ICV and regional brain volume is consistent across multiple brain regions^20,24,29^. Moreover, this proportionality is particularly uncertain for volumes within the brainstem, which is our target region. Thus, we first investigated the relationship between ICV and target ROI volumes in normal healthy subjects. The scatterplots of ICV and ROI volume showed that the y-intercepts of the regression lines were positive for all investigated ROIs (Fig. 1), which indicated that the two measures are not proportional. These results justify the more rigorous volume adjustment applied in our current study.

We confirmed the presence of widespread brain volume loss in patients with DAI. The percentage of volume reduction in the cerebrum was estimated to be 7.4% on average. The percent loss was marked in the brainstem (13.9% on average), which suggests that the brainstem is one of the most affected brain regions in DAI pathology. This result is consistent with postmortem histopathological studies that have reported that the brainstem, particularly the upper brainstem, is one of the major locations of axonal injury^2,8–11^. Indeed, a biomechanical model has demonstrated that the greatest strain effects occur in the brainstem, particularly in the upper brainstem^30,31^.

The association between acute-phase consciousness disorder and brainstem injury has been explored previously in patients with TBI. The existence of lesions inside the brainstem, particularly in the midbrain or pons, has been reported to be associated with the duration of loss of consciousness^13,32^. However, another study concluded that the duration of PTA is not associated with lesions in any specific brain region^33^. Previous studies investigating the relationship between acute loss of consciousness and brainstem injury included a wide range of TBI subjects, and thus many of these patients likely had mixed FI and DAI pathology. In contrast, to the best of our knowledge, our study is the first to investigate this issue in subjects with predominantly DAI pathology.

Our investigation of DAI had some shortcomings that should be considered when interpreting the findings. Among the various types of TBI severity indicators, we used the duration of PTA because it was the only indicator obtained for every DAI participant. The duration of PTA is seldom assessed prospectively using a clinical scale in the acute clinical care setting in Japan; this applied to the DAI subjects in this study. Because of the paucity of cases in which PTA was prospectively assessed in the acute phase, the duration of PTA was estimated retrospectively. To assess PTA duration using the collected acute-phase information as objectively and consistently as possible, we applied criteria based on orientation to person, time, and place and continuous memory for day-to-day events, as described in the Materials and methods. These criteria were developed with reference to the Westmead Post-Traumatic Amnesia Scale (WPTAS).

To avoid underestimation of the PTA duration, we calculated it at the weekly rather than daily scale. Therefore, the PTA duration may have been overestimated in some cases. When a system for classifying TBI severity as severe, moderate or mild^34^ was applied to the DAI patients in this study, six of the seven patients with mild or moderate injuries according to the GCS or JCS score were classified as severe. This may reflect overestimation of the PTA. It might be preferable to avoid application of a retrospectively estimated PTA duration when classifying TBI severity. Nevertheless, provided that the PTA duration is determined in a consistent manner on the basis of specific criteria, it can serve as a “yardstick” of injury severity even if the duration is overestimated. Therefore, we believe that the retrospectively estimated PTA durations were not problematic with respect to the results of the correlation analysis. The significant correlation between PTA duration and brainstem volume (particularly the pons and midbrain) observed in our DAI patients demonstrate that early phase consciousness disorders, as indexed by the duration of PTA, affect the chronic-phase brainstem volume in this group.

This study had several limitations. First, although the reliability of our model was excellent for the cerebrum (ICC = 0.92) it was only fair-to-good for the whole brainstem and subfields thereof (ICC = 0.5–0.8). The size of the cranium is one of the main determinants of regional brain volumes. Thus, we adjusted the raw volume according to the ICV. However, the relationship between the shape of the cranium and regional brain volumes is likely more complex. In future studies, modeling the inner surface of the cranium as a three-dimensional structure may increase model reliability. Moreover, because of racial differences in cranial and brain morphology^35–37^, the model equations established in this study are currently applicable only to Japanese and other East Asian adults. However, we believe that our observation and analysis method could be applied to other races to derive equations for estimating normal healthy brain volumes. Therefore, our novel method has potential as a universal, generalizable approach for estimating healthy brain volumes. As a further study limitation, PTA could not be assessed prospectively in our DAI patients, which limited assessment accuracy and might have led to overestimation of its duration. Although our results suggest an association between acute-phase consciousness disorders and chronic-phase brainstem atrophy, our method cannot directly reveal the pathophysiology of consciousness disorders in TBI subjects with DAI. Furthermore, the DAI group was relatively small and the time since injury varied among the patients. To further explore the issues addressed in this study, longitudinal studies collecting comprehensive data on cognitive and behavioral impairments are needed.

In conclusion, we established a model for estimating the healthy volume of the brainstem in individuals without brain injury according to age, sex, and the ICV. We then confirmed brainstem volume reduction in patients with DAI. Finally, we demonstrated that brainstem volume was associated with the duration of PTA, which is an early indicator of injury severity. Our findings suggest that the severity of acute-phase consciousness disorder, which is quantified according to the duration of PTA, is among the factors affecting the chronic-phase volume of the brainstem in patients with DAI. These results could facilitate clinical assessment of DAI.

## Materials and methods

We organized this study as a combination of two different experiments, both of which used a cross-sectional approach.

### Experiment 1

#### Participants

The healthy participants were paid volunteers recruited from the local community through advertisements (posters and websites), word of mouth, direct approach, and an agency. Many of the participants were students, staff at Kyoto University, and their relatives and friends. Their data were stored in a database that is used to recruit subjects to neuroimaging studies conducted in the Department of Psychiatry, Kyoto University. We used the data of 232 healthy Japanese/other East Asian adults aged 18–68 years who were recruited from April 2014 to June 2018, and randomly divided them into two age-controlled groups at a 4:1 ratio using the permuted block method (*n* = 185 and *n* = 47). Three men from the larger group were excluded from the analysis because of image processing errors. The two groups were named Group A (*n* = 182, 99 men, age range: 18–64 years, median age: 33 years) and Group B (*n* = 47, 24 men, age range: 20–68 years, median age: 34 years; Table 1). Group A was used to create the model for estimating each healthy regional volume, and Group B was used to test the validity of the model.

#### MRI

All participants underwent MRI scanning on a 3 T whole-body scanner with a 40 mT/m gradient and a receiver-only 32-channel phased-array head coil (MAGNETOM Tim Trio, Siemens, Erlangen, Germany). The scanning parameters of the T1-weighted three-dimensional magnetization-prepared rapid gradient-echo sequence were as follows: repetition time = 2000 ms; echo time = 3.4 ms; inversion time = 990 ms; field of view = 225 × 240 mm; matrix = 240 × 256; resolution = 0.9375 × 0.9375 × 1.0 mm^3^; bandwidth = 130 Hz/pixel; flip angle = 8°; and 208 axial slices without intersection gaps.

#### Image processing and measurement of regional brain volumes

T1-weighted images were visually checked and processed using the semi-automatic processing pipeline, recon-all, of FreeSurfer software version 6.0 (http://surfer.nmr.mgh.harvard.edu/) for volumetric segmentation and cortical reconstruction. FreeSurfer processing included the removal of non-brain tissue, automated Talairach transformation, segmentation of subcortical white matter and deep gray matter volumetric structures, intensity normalization, tessellation of the gray matter/white matter boundary, automated topology correction, and surface deformation^38–40^. Estimated total intracranial volume, which is also referred to as the ICV, was output by FreeSurfer (as “aseg.stats” files) for each subject. Rather than segmentation, the ICV is computed on the basis of an atlas scaling factor, which is the determinant of an affine transformation matrix. This matrix is used to align the image with the atlas (http://surfer.nmr.mgh.harvard.edu/fswiki/eTIV)^41^The ICV values derived by FreeSurfer were in good agreement with those obtained through ICV reference segmentation^25^. The cortical gray matter, cerebral white matter, and subcortical gray matter volumes were also output as “aseg.stats” files for each subject and used in subsequent analyses. After manually checking the segmented view to ensure that the “cortical gray matter,” “subcortical gray matter,” and “cerebral white matter” were mutually independent areas that constituted almost all of what clinicians would generally recognize as the cerebrum on brain images, we defined the combination of these three regions as the “cerebrum” and the volume of the cerebrum as the sum of the volumes of these three regions. This continuous area of the cerebrum included the thalamus, caudate, putamen, pallidum, hippocampus, and amygdala but not the cerebellum or brainstem. We also ran the FreeSurfer extension pipeline designed for brainstem subfield segmentation^42^, and the volumes of the whole brainstem and its four subdivisions (i.e., the medulla, pons, midbrain, and superior cerebellar peduncle) were output as “brainstemSsVolumes.v10.txt” files for each subject. All segmented images were checked visually for quality. The superior cerebellar peduncle volume was not used for the analysis because it is a residual-like minor subfield that accounts for less than 3% of the volume of the whole brainstem. Thus, the brain ROIs that were used to investigate volume were as follows: (1) the whole brainstem, (2) medulla, (3) pons, (4) midbrain, and (5) cerebrum. Example coronal and sagittal ROIs are illustrated in Supplementary Fig. 1.

#### Statistical analysis

We first checked the relationships between each ROI volume and its potential explanatory variables using the volumetric measures in healthy individuals from Group A. Pearson’s correlation analysis was performed to assess the association between each ROI volume and age or ICV. After identifying that ICV was the major determinant of the volume of any ROI, two-way ANOVA was performed to check the interaction between sex and ICV for each ROI volume. A regression equation obtained from the regression analysis (described below) was used for the model to estimate the ENHV of each ROI from “ICV” or “ICV and age.” For any ROI with a significant interaction between sex and ICV, a regression analysis was performed on each sex subgroup separately, and the regression equation for each sex subgroup was incorporated into the ROI’s model equation with a dummy variable representing sex. For any ROI without a significant interaction, a regression analysis was performed on the whole sample of Group A (males and females together), and the regression equation was readily employed as the ROI’s model equation. In this regression analysis, if either or both “ICV” and “age” showed a significant correlation with the regional volume, they were set as the independent variable(s), and the volume of each ROI was set as the dependent variable.

Before applying our novel model to DAI patients, we tested its validity. Using volumetric data for Group B (i.e., the measured volumes and corresponding model-estimated ENHVs), ICCs were calculated for all ROIs using a two-way mixed-effects model for single measures (absolute agreement). The ICC values were classified as follows: < 0.4, poor model reliability; 0.4–0.59, fair reliability; 0.6–0.74, good reliability; and 0.75–1.0, excellent reliability. In this study, the threshold for regression-based equation validity was set as ICC > 0.4, in accordance with published guidelines^43^.

### Experiment 2

#### Participants

Twenty-two Japanese patients clinically diagnosed with DAI were recruited from the outpatient clinic of the Neuropsychology Unit at the Department of Psychiatry and Neurosurgery, Kyoto University Hospital from December 2014 to January 2019. All patients underwent computed tomography during the early presentation of trauma. High-contrast fluid-attenuated inversion recovery imaging was used to assess DAI. Diagnoses were confirmed by consensus among three expert neuropsychiatrists (G.F., T.M., and K.U.) according to the following inclusion criteria: (1) brain injury sustained through significant head trauma at least 3 months before enrollment; (2) loss of consciousness at the time of injury and PTA duration of ≥ 1 day; (3) acute imaging findings of head trauma, including microhemorrhages, in the deep white matter; (4) brain MRI showing possible diffuse pathology and an absence of focal parenchymal lesions (e.g., lobar contusions and hematoma/hemorrhage > 2 mm diameter); and (5) aged over 18 years. The exclusion criteria were as follows: (1) history of neurological or psychiatric disorders before TBI; (2) history of drug abuse; (3) history of another TBI; or (4) visual or visuoperceptual deficits. These criteria, which are effective for eliminating FI factors and extracting DAI effects, are similar to those used in previous studies by our group^44–47^. In 21 DAI patients who underwent susceptibility-weighted imaging, we classified hemorrhagic lesions using the extended anatomical grading for DAI^48^.

The GCS or JCS score was obtained for all 18 DAI subjects (from the hospital where acute care was provided) and used for classification of TBI as severe, moderate, or mild, as in previous studies^34,49,50^.

For each DAI subject, the PTA duration was retrospectively assessed and included in the analysis as an indicator of acute-phase injury severity. It corresponded to the time between the TBI and week in which orientation and continuous memory for day-to-day events had definitely returned. Hospital records compiled by medical professionals, along with the detailed accounts and diaries of the subjects and their relatives, were collected as reliable acute-/subacute-phase reference information to enable assessment of orientation and memory. On the basis of this information, the duration of PTA (in weeks) was determined as the length of time since the TBI needed for the patient to meet the following criteria: (1) the patient can state their age and date of birth correctly (orientation to person); (2) the patient can state the year, month, date, day of the week, and time of day (morning, afternoon, or night) correctly (orientation to time); (3) the patient can state the name of the city and building that they are in correctly (orientation to place); and (4) the patient shows no signs of an inability to lay down memories from one day to the next (normal continuous memory). The retrospective measurement of PTA duration has been demonstrated to be valid and correlate highly with the prospective assessment of PTA duration^51^. The determination of the duration of PTA was double-checked by G.F. and K.U. to ensure accuracy.

A subsample of 60 adult HCs (47 males and 13 females) were randomly selected from among the 229 healthy participants (123 men and 106 women) in Groups A and B in Experiment 1, and were matched with the 22 DAI participants (17 men and 5 women) according to sex and age (Table 5). For the 229 healthy participants, a random number between 0 and 1 was output using Excel 2016 software (Microsoft Corp., Redmond, WA, USA); the 47 males and 13 females with the highest numbers were included in the subsample.

#### MRI

The same as Experiment 1*.*

#### Image processing and measurement of regional brain volumes

The same as Experiment 1*.*

#### Statistical analysis

The Mann–Whitney U test was performed to compare the volumes of the cerebrum or the brainstem and its three subdivisions (i.e., the medulla, pons, and midbrain) between 22 patients with DAI and 60 HCs. Next, for each DAI and HC individual, the ENHV at the time of the MRI scan of each ROI for which the validity of the model was ensured was estimated using our model formula. The measured volume was expressed as a percentage of its corresponding ENHV according to Eq. (4), which we defined as the “ENHV-adjusted volume”.

4 $${\text{ENHV-adjusted\,volume }}\left( {\text{percent\,of\,ENHV}} \right) = \frac{{\text{measured\,volume }}}{{{\text{ENHV }}\left( {\text{estimated\,by\,the\,model}} \right){ }}} \times 100$$

To compare the ENHV-adjusted volumes of DAI subjects, related-samples Wilcoxon signed-rank tests were performed between the whole brainstem and the cerebrum, and Friedman tests and related-samples Wilcoxon signed-rank tests were performed among the subfields of the brainstem. Furthermore, to assess the association between the duration of PTA and the ENHV-adjusted volume of each ROI, removing the potential effect of time since injury, partial correlation analyses, controlling for time since injury, were performed. In cases where the ENHV-adjusted volume was replaced with the ICV-corrected volume based on the conventional proportions method, the same partial correlation analyses were performed. All comparison tests were two-tailed.

All statistical analyses were performed using Excel 2016 (Microsoft Corp.) and SPSS 23 (IBM Corp., Armonk, NY, USA).

This study was approved by the Committee on Medical Ethics of Kyoto University and conducted in accordance with the Code of Ethics of the World Medical Association. Written informed consent was obtained from all participants, and the study was conducted in accordance with the Declaration of Helsinki.

## Supplementary Information

Supplementary Information.

## Data Availability

The data that support the findings of this study are available from the corresponding author upon reasonable request.
