# Supplementary material for: A model for estimating the brainstem volume in normal healthy individuals and its application to diffuse axonal injury patients
Source: Sci Rep. 2023 Jan 2;13:33. doi: 10.1038/s41598-022-27202-x (PMC9807567; doi:10.1038/s41598-022-27202-x)

**Supplementary Materials**

**manuscript ID** 3adfa88a-6206-400d-a24d-9c90b0347126

**Title**: A model for estimating the brainstem volume in normal healthy individuals and its application to diffuse axonal injury patients

**Gaku Fujimoto^1,*^, Shiho Ubukata^1,2^, Genichi Sugihara^3^, Naoya Oishi^2^, Toshihiko Aso^1,4^, Toshiya Murai^1^, and Keita Ueda^1,5^**

1 Department of Psychiatry, Graduate School of Medicine, Kyoto University, 54 Shogoin-Kawahara-cho, Sakyo-ku, Kyoto 606-8507, Japan

2 Medical Innovation Center, Graduate School of Medicine, Kyoto University, 53 Shogoin-Kawahara-cho, Sakyo-ku, Kyoto 606-8507, Japan

3 Department of Psychiatry and Behavioral Sciences, Graduate School of Medical and Dental Sciences, Tokyo Medical and Dental University, 1-5-45 Yushima, Bunkyo-ku, Tokyo 113-8510, Japan

4 Laboratory for Brain Connectomics Imaging, RIKEN Center for Biosystems Dynamics Research, 6-7-3 Minatojima-minamimachi, Chuo-ku, Kobe, Hyogo 650-0047, Japan

5 Department of Medical Welfare, Faculty of Health Sciences, Kyoto Koka Women's University, 38 Nishikyogoku Kadono-cho, Ukyo-ku, Kyoto 615-0882, Japan

*mtfuji1@kuhp.kyoto-u.ac.jp

**Supplementary Table 1 Results of regression analysis for each region of interest by sex**

| **ROI** | **Sex and number of subjects** | **Explanatory variable(s)** | **R square (adjusted R square)** | **Significance F** |
| --- | --- | --- | --- | --- |
| Medulla | 99 males and 83 females | ICV | 0.296(0.241) | 1.12E-12 |
| Whole brainstem | 99 males | ICV | 0.270 (0.262) | 3.53E-08 |
| Whole brainstem | 83 females | ICV | 0.133 (0.122) | 6.94E-04 |
| Pons | 99 males | ICV | 0.235 (0.227) | 3.79E-07 |
| Pons | 83 females | ICV | 0.086 (0.075) | 7.17E-03 |
| Midbrain | 99 males | ICV | 0.312 (0.305) | 1.86E-09 |
| Midbrain | 83 females | ICV | 0.250 (0.241) | 1.47E-06 |
| Cerebrum | 99 males | ICV and age | 0.656 (0.648) | 5.98E-23 |
| Cerebrum | 83 females | ICV and age | 0.616 (0.607) | 2.25E-17 |

ROI, region of interest; ICV, intracranial volume

**Supplementary Table 2 Clinical characteristics of the DAI patients**

| **Subject ID** | **Sex** | **Age (years)** | **Time since injury (months)** | **GCS score** | **JCS score** | **Injury severity by GCS or JCS** | **PTA**^a^ **(weeks)** | **Cause of injury** | **Anatomical grading for DAI**^b^ |
| --- | --- | --- | --- | --- | --- | --- | --- | --- | --- |
| DAI1 | Male | 27 | 131 | 6 | NA | Severe | 13 | Traffic accident | I |
| DAI2 | Male | 28 | 112 | NA | 100 | Severe | 9 | Traffic accident | II |
| DAI3 | Male | 48 | 355 | NA | NA | NA | 6 | Traffic accident | I |
| DAI4 | Male | 21 | 16 | 10 | NA | Moderate | 2 | Traffic accident | II |
| DAI5 | Male | 23 | 39 | NA | 200 | Severe | 22 | Traffic accident | IV |
| DAI6 | Female | 32 | 64 | 5 | NA | Severe | 3 | Traffic accident | II |
| DAI7 | Male | 31 | 169 | NA | 300 | Severe | 3 | Fall | III |
| DAI8 | Male | 25 | 105 | NA | 300 | Severe | 11 | Traffic accident | III |
| DAI9 | Female | 47 | 160 | NA | NA | NA | 9 | Traffic accident | 0 |
| DAI10 | Female | 41 | 6 | 15 | NA | Mild | 2 | Traffic accident | II |
| DAI11 | Male | 46 | 79 | 15 | NA | Mild | 1 | Traffic accident | 0 |
| DAI12 | Male | 50 | 131 | NA | 200 | Severe | 5 | Traffic accident | I |
| DAI13 | Male | 61 | 25 | 7 | NA | Severe | 9 | Traffic accident | III |
| DAI14 | Male | 32 | 19 | 14 | NA | Mild | 9 | Traffic accident | IV |
| DAI15 | Male | 33 | 165 | 4 | NA | Severe | 9 | Traffic accident | NA |
| DAI16 | Female | 41 | 42 | 11 | NA | Moderate | 2 | Traffic accident | I |
| DAI17 | Male | 50 | 5 | NA | 2 | Mild | 2 | Traffic accident | I |
| DAI18 | Male | 57 | 17 | NA | NA | NA | 9 | Traffic accident | 0 |
| DAI19 | Female | 39 | 276 | NA | 300 | Severe | 26 | Traffic accident | 0 |
| DAI20 | Male | 22 | 38 | NA | NA | NA | 1 | Traffic accident | I |
| DAI21 | Male | 25 | 3 | 13 | NA | Mild | 3 | Traffic accident | I |
| DAI22 | Male | 53 | 5 | 3 | NA | Severe | 13 | Traffic accident | I |

^a^“PTA (weeks) = X” indicates “7X–6 ≤ PTA (days) ≤ 7X”. For example, “PTA (weeks) = 2” indicates “PTA (days) = 8–14”.

^b^Stage I, hemispheric lesions; stage II, hemispheric and additional corpus callosum lesions; stage III, brainstem lesions; stage IV, lesions in the substantia nigra or mesencephalic tegmentum; stage 0, absence of hemorrhagic spots in chronic-phase images but evidence of microbleeds on acute-phase X-ray computed tomography images

DAI, diffuse axonal injury; GCS, Glasgow Coma Scale; JCS, Japan Coma Scale; PTA, duration of posttraumatic amnesia; NA, not available

**Supplementary Table 3** **Results of partial correlation analysis between the expected normal healthy volume-adjusted volume and the duration of posttraumatic amnesia in 22 diffuse axonal injury patients (controlling for time since injury)**

|  | **Partial correlation coefficient** | **P-value** |
| --- | --- | --- |
| **Whole brainstem** | −0.449* | 0.041 |
| **Medulla** | −0.216 | 0.347 |
| **Pons** | −0.459* | 0.036 |
| **Midbrain** | −0.502* | 0.020 |
| **Cerebrum** | −0.251 | 0.273 |

*Significant at P < 0.05 (uncorrected)

**Supplementary Figure 1 Example coronal and sagittal regions of interest**


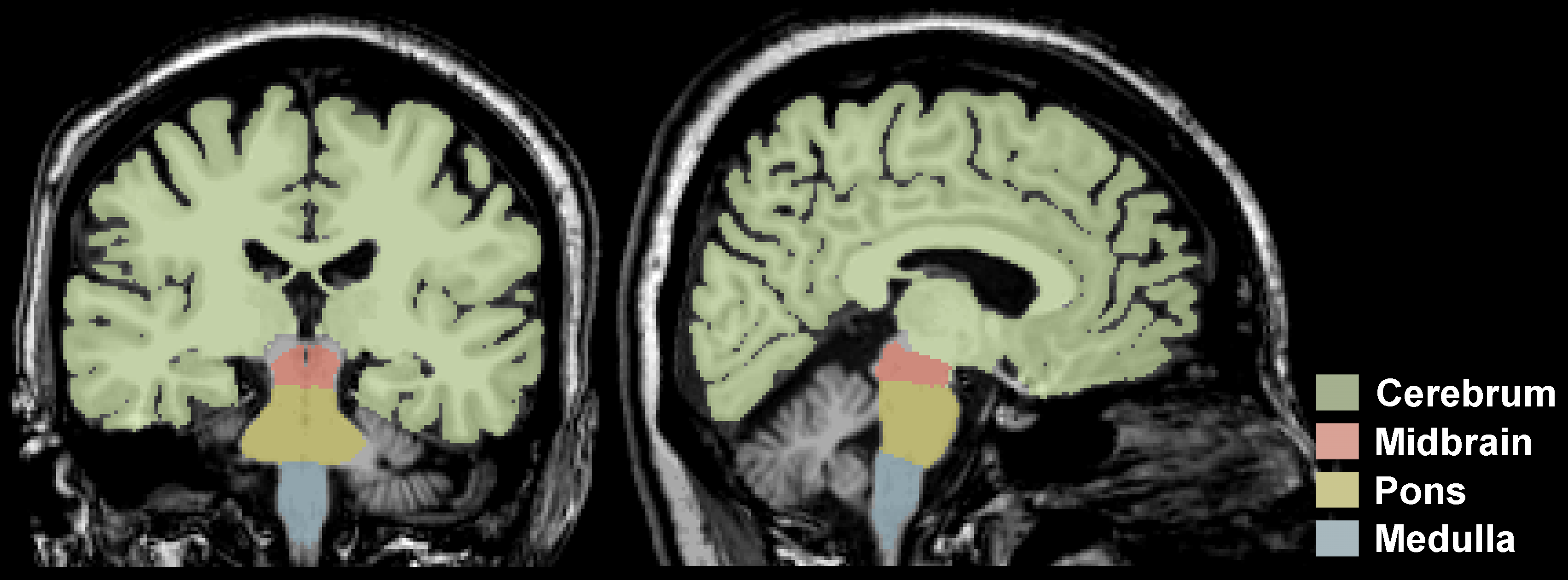

Supplement: Supplementary file 1 — Supplementary Information. [file 41598_2022_27202_MOESM1_ESM.docx]
